# Apolipoprotein E and Apolipoprotein E receptor 2 signaling as drug targets in Alzheimer’s disease

**DOI:** 10.1002/alz.086014

**Published:** 2025-01-03

**Authors:** Abdel Ali Belaidi, Ashley I. Bush, Scott Ayton

**Affiliations:** ^1^ The Florey Institute of Neuroscience and Mental Health, The University of Melbourne, Australia, Melbourne, VIC Australia

## Abstract

**Background:**

Allelic variation in apolipoprotein E (APOE) is by far the greatest contributor to Alzheimer’s disease (AD) after age, but the mechanisms underlying how APOE impacts on the pathology of AD remain undefined. While most research is focusing on mechanisms associated with the presence of the *APOE* risk allele, several aspects of APOE biology remain poorly understood. In particular, the physiological relevance of APOE receptors and their impact on disease progression have been overlooked.

**Method:**

Ferroptosis was induced in N27 neurons using multiple agents (erastin, RSL3) and toxicity was evaluated using different viability assays (MTT, Propidium iodide, LDH) and ferroptosis. N27 neurons were transfected with a plasmid encoding APOER2‐GFP fusion protein or a GFP‐expressing control vector, while recombinantly expressed and purified APOE was used as an APOE source.

**Result:**

Here, we present data supporting a function of APOE in tandem with APOER2 in protecting neurons from ferroptosis. Ferroptosis is a cell death pathway characterized by the accumulation of lethal lipid hydroperoxides resulting from iron‐dependent oxidation of polyunsaturated fatty acids. APOER2 expression in neurons resulted in increase in glutathione peroxidase 4 (GPX4) expression, which functions by converting toxic lipid peroxides into non‐toxic lipid alcohol and thus increases resistance to ferroptosis. We further report that APOER2 expression potentiates APOE‐mediated protection from ferroptotic cell death in in N27 neurons. Further mechanistic studies are ongoing to validate the underlying mechanism and the involved signaling cascades.

**Conclusion:**

We present evidence for the involvement of APOE and APOER2 in neuronal protection from ferroptotic cell death. These findings represent new drug targets that can be explored to slowdown neurodegeneration in AD.

**Funding**: This study was supported by grants from the Alzheimer’s Association and National Health & Medical Research Council of Australia.

